# Fatty Acid Oxidation Promotes Cardiomyocyte Proliferation Rate but Does Not Change Cardiomyocyte Number in Infant Mice

**DOI:** 10.3389/fcell.2019.00042

**Published:** 2019-03-22

**Authors:** Tongtong Cao, Daniela Liccardo, Ryan LaCanna, Xiaoying Zhang, Rong Lu, Brian N. Finck, Tani Leigh, Xiongwen Chen, Konstantinos Drosatos, Ying Tian

**Affiliations:** ^1^Department of Pharmacology, Center for Translational Medicine, Lewis Katz School of Medicine, Temple University, Philadelphia, PA, United States; ^2^Department of Pathology, Shanghai University of Traditional Chinese Medicine, Shanghai, China; ^3^Department of Physiology, Cardiovascular Research Center, Lewis Katz School of Medicine, Temple University, Philadelphia, PA, United States; ^4^Division of Geriatrics and Nutritional Sciences, Department of Medicine, Washington University School of Medicine in St. Louis, St. Louis, MO, United States

**Keywords:** fatty acid oxidation, cardiomyocyte, proliferation, hypertrophic growth, infant mice

## Abstract

Cardiomyocyte proliferation accounts for the increase of cardiac muscle during fetal mammalian heart development. Shortly after birth, cardiomyocyte transits from hyperplasia to hypertrophic growth. Here, we have investigated the role of fatty acid β-oxidation in cardiomyocyte proliferation and hypertrophic growth during early postnatal life in mice. A transient wave of increased cell cycle activity of cardiomyocyte was observed between postnatal day 3 and 5, that proceeded as cardiomyocyte hypertrophic growth and maturation. Assessment of cardiomyocyte metabolism in neonatal mouse heart revealed a myocardial metabolic shift from glycolysis to fatty acid β-oxidation that coincided with the burst of cardiomyocyte cell cycle reactivation and hypertrophic growth. Inhibition of fatty acid β-oxidation metabolism in infant mouse heart delayed cardiomyocyte cell cycle exit, hypertrophic growth and maturation. By contrast, pharmacologic and genetic activation of PPARα, a major regulator of cardiac fatty acid metabolism, induced fatty acid β-oxidation and initially promoted cardiomyocyte proliferation rate in infant mice. As the cell cycle proceeded, activation of PPARα-mediated fatty acid β-oxidation promoted cardiomyocytes hypertrophic growth and maturation, which led to cell cycle exit. As a consequence, activation of PPARα-mediated fatty acid β-oxidation did not alter the total number of cardiomyocytes in infant mice. These findings indicate a unique role of fatty acid β-oxidation in regulating cardiomyocyte proliferation and hypertrophic growth in infant mice.

## Introduction

Cardiac myocytes constitute the most fundamental functional units of the heart. Mammalian cardiomyocytes proliferate rapidly and use glycolysis as the main source of energy during fetal life (Lopaschuk et al., 1991; Soonpaa et al., 1996; Li et al., 1997b). After birth, cardiomyocytes exit cell cycle and transit from hyperplasia to hypertrophic growth (Li et al., 1997a). Over the first week of postnatal life in mice, cardiomyocytes exhibit a transient wave of increased cell cycle activity (Lopaschuk et al., 1991; Soonpaa et al., 1996; Li et al., 1997a,b), that proceeds as cardiomyocyte binucleation, cell size enlargement and a metabolic switch from glycolysis to fatty acid β-oxidation (Lopaschuk and Spafford, 1990; Li et al., 1997a; Makinde et al., 1998; Ahuja et al., 2007; Naqvi et al., 2009; Lopaschuk and Jaswal, 2010; Zebrowski and Engel, 2013). However, it remains unknown whether the switch in metabolic pathways controls cardiomyocyte proliferation and hypertrophic growth.

There is a growing appreciation that metabolic signals are integrated to cell cycle progression. In relatively simpler cellular systems grown in tissue culture conditions where nutrient supply could be tightly regulated, it is well established that cell cycle progression is influenced by metabolites or a combination of metabolites and an oscillating signal (Tu et al., 2007; Buchakjian and Kornbluth, 2010; Mandal et al., 2010; Lee and Finkel, 2013; Shi and Tu, 2013; Yalcin et al., 2014; Papagiannakis et al., 2017). For instance, studies in the growth of budding yeast show that metabolic intermediate, acetyl-CoA, controls G1 phase progression. Subsequent analysis of gene expression from chromatin immunoprecipitation assay revealed that acetyl-CoA induces the acetylation of histone in the growth genes and enables cell growth and proliferation (Cai et al., 2011; Shi and Tu, 2013). Relevant studies, using interventions that alter nutrient utilization, show that oscillating metabolism of budding yeast determines the timing of the cell-cycle phases and sets the pace of cell division (Papagiannakis et al., 2017).

By contrast, little is known about whether and how metabolic state of cardiomyocyte influences cell growth and division in the complex mammalian heart. Cardiomyocytes in fetal mouse hearts can proliferate quite dramatically and exit cell cycle soon after birth (Soonpaa et al., 1996; Lopaschuk and Jaswal, 2010). Recent studies show that the heart exhibits a transient phase of cardiomyocyte cell cycle reactivation during early postnatal life (Soonpaa et al., 1996). However, postnatal cell cycle progression has several unique features compared to that of fetal life. First, rather than generating mononucleated cardiomyocytes, postnatal cardiomyocytes failed to complete cell division, which results in the formation of binucleated cardiomyocytes (Soonpaa et al., 1996; Li et al., 1997b). Second, instead of maintaining constant cell volume, postnatal cardiomyocytes exhibit hypertrophic growth (Li et al., 1996). Cardiomyocyte binucleation seems to be closely related with the onset of hypertrophy. Third, unlike proliferating cardiomyocyte that use glycolysis as a source of energy in fetal heart, fatty acid β-oxidation is the primary catabolic pathway that provides energy needed for contractile function in early postnatal hearts (Lopaschuk and Jaswal, 2010). Although a metabolic shift from glycolysis to fatty acid oxidation has been described during the first week of postnatal heart development in mice, it is unclear whether this metabolic change accounts for cardiomyocyte proliferation and hypertrophy or is simply a consequence of increased O_2_ availability that coincides with transition from fetal to postnatal life.

In this study, we investigated the cardiac metabolic state in the developing cardiomyocyte during early postnatal life in mice, and the role of fatty acid β-oxidation in cardiomyocyte proliferation and the transition from hyperplasia to hypertrophy. In doing so, we make important insights about the biological consequences of alterations in energy metabolism on the growth of cardiac muscle.

## Materials and Methods

### Animal Studies

CD-1^®^ IGS mice were purchased from Charles River Laboratories (Horsham, PA, United States). Generation and genotyping of the αMHC-PPARα line has been previously described (Finck et al., 2002). All litter sizes were adjusted as 8–10 pups per litter. The sex of infant mice used in these studies was not determined. In general, sample size was chosen to use the least number of animals to achieve statistical significance and no statistical methods were used to predetermine sample size. Animals were allocated to experimental groups based on genotype and we did not use exclusion, randomization or blinding approaches. All the animal experiments were performed according to the NIH guidelines (Guide for the care and use of laboratory animals). All experimental procedures involving animals in this study were reviewed and approved by Temple University Medical Center’s Institutional Animal Care and Use Committee.

### Neonatal Cardiomyocyte Isolation and Culture

Mouse neonates (<10 days of age) were sacrificed by decapitation. Cardiomyocytes were collected using previously described protocol (Tian et al., 2015). Briefly, mouse cardiomyocytes were isolated by enzymatic disassociation of one day-old neonate hearts (P1). Cells were plated differentially for 2 h to remove fibroblasts. Cardiomyocytes were plated on laminin-coated (10 μg/cm^2^) 96-well plates at 1.5 × 10^4^ cells per well. On the following day, culture medium was replaced with fresh medium (Opti-MEM supplemented with 10% fetal bovine serum, 5% horse serum and 10 Unit/ml Penicillin-Streptomycin) either with or without etomoxir (5 μM).

### Immunostaining for cTnT in Cardiomyocytes and Myocyte Size Measurements

Isolated cells were fixed by 4% PFA at room temperature (r.t.) for 15 min. Fixed cells were pelleted, washed two times in 1 ml of PBS, then re-suspended in permeabilization buffer at r.t. for another 15 min. Permeabilized cells were pelleted, washed two times in 1 ml of PBS, then processed for blocking and immunostaining for cardiomyocyte marker (cTnT, 1:200; Thermo Fisher Scientific, MS-295-P1). DAPI was used to counterstain nuclei. Images were acquired for analysis within 48 h. Cell length and width were determined from phase contrast images using the ImageJ plugin Coli-Inspector. Length and width measurements from ≥ 300 cells from ≥ 3 biological replicates were used to generate data for each condition.

### Histology

Neonatal and adult heart tissues were fixed in 2 and 4% formaldehyde individually and processed for paraffin histology and sectioned using routine procedures. Immunohistochemical staining was performed using previously described protocol (Tian et al., 2015). Primary antibodies are: Ki67 (1:50; Abcam, ab16667), Phospho-Histone H3 (PH3, 1:200; Cell Signaling Technology; 9706L), Aurora B kinase (1:200; BD Transduction Laboratories; 611082), cardiac Troponin T (cTnT, 1:100; Thermo Fisher Scientific, MS-295-P1), cardiac Troponin I (cTnI, 1:200; Abcam; ab47003), Caveolin-1 (1:100, Cell Signaling Technology; 3238S), Wheat Germ Agglutinin (WGA), Alexa Fluor^®^ 633 Conjugate was used on the same sections to outline cardiomyocytes. DAPI was used to counterstain nuclei. Apoptosis was measured using *in situ* Cell Death Detection Kit (Roche). Cell proliferation was measured using Click-iT^®^ EdU (5-ethynyl-2′-deoxyuridine) Alexa Fluor^®^ Imaging Kit (Thermo Fisher Scientific). The slides were imaged and subjected to an independent blinded analysis, using a Zeiss LSM 710 confocal microscope and ImageJ software. Images shown are representative view of multiple fields from at least four independent samples per group. Quantitation of cell numbers was done using images acquired on confocal microscopy and the ImageJ with the “Cell Counter” plug-in, counting multiple fields from at least 4 independent samples per group and about 2200–5000 cTnT+ cells per sample.

### *In vivo* Treatment and EdU Labeling

Infant mice were treated with etomoxir (15 μg/g/day; Sigma, E1905) or GW7647 (2 μg/g/day; Sigma, G6793) or saline via intraperitoneal (i.p.) injection on postnatal day 2 (P2), P3 and P4, one dose per day. For EdU labeling, infant mice were injected with one dose of EdU 50 mg/kg via intraperitoneal injection and sacrificed after 3 h.

### Extracellular Flux Measurements

Metabolic profiling was assessed performing glycolytic stress test and palmitate oxidation test using a Seahorse XF flux analyzer 96. Cardiomyocytes were isolated from 8 to 10 infant mice on day 2 (P2), 3(P3), 5 (P5), and 7 (P7) after birth. Cells were seeded on Seahorse XF-96 plates coated with laminin at a density of 4 × 10^4^ cells/well and incubated for 24 h in culture cells media. One day prior to the experiment, sensor cartridges were hydrated with XF calibrate solution (pH 7.4) and incubated at 37°C in a non-CO_2_ incubator for 24 h. To evaluate glycolytic function, culture medium was exchanged with the XF Assay media (XF-base media supplemented with 2 mM glutamine, pH7.4) and the microplates placed into a 37°C non-CO_2_ incubator for 1 h prior to the start of an assay. Extracellular acidification rate (ECAR) was measured at baseline and after the injection of glucose (10 mM), oligomycin (1 μM) and 2-deoxyglucose (2-DG, 50 mM).

To evaluate the effect of etomoxir and GW7647 on cardiomyocyte glycolytic function, we plated cardiomyocytes isolated from P3 infant mice in the presence of etomoxir (5 μM) or GW7647 (2 μM) for 24 h. On the next day, media was replaced with XF Glycolysis Assay media and ECAR levels were measured before and after the injection of Glucose (10 mM).

To assess fatty acid oxidation, endogenous substrates within the cells were depleted replacing the culture media with Substrate-Limited Media (D-MEM supplemented with 0.5 mM Glucose, 1 mM GlutaMAX, 0.5 mM carnitine and 1% FBS) and incubating the cells for an additional 24 h. One hour prior to the assay, culture media was replaced to FAO assay media (KHB supplemented with 2.5 mM glucose, 0.5 mM carnitine and 5 mM Hepes, pH was adjusted to 7.4). Oxygen consumption rate (OCR) was measured at baseline and after the injection of saturating amount of Palmitate-BSA (XF palmitate–BSA FAO substrate, Seahorse bioscience, Agilent Technology) and two doses of etomoxir (40 μM) to obtain the maximal inhibition of exogenous Fatty acid oxidation. To assess glucose oxidation, 1 h prior to the assay, culture media was replaced to substrate-free XF- Base media. OCR levels were measured at baseline and after the injection of Glucose 10 mM and oligomycin (2 μM). Three baseline measurements of ECAR and OCR were taken before glucose or palmitate-BSA injection, and 3 response measurements were taken after the addition of each other compound. ECAR and OCR were expressed as a percentage of the baseline measurement. Glycolysis was quantified as the maximum percentage increase of ECAR over baseline, after the injection of saturating amount of glucose. Glycolytic capacity defined as maximum obtainable glycolysis after inhibition of mitochondrial ATP production was measured as maximum percentage increase over baseline after oligomycin injection. Glucose oxidation was measured as maximum percentage increase over baseline after glucose and oligomycin injection. β-oxidation was evaluated as maximum percentage increase over baseline after palmitate-BSA injection.

### Echocardiography

Mice were anesthetized with inhalation of isoflurane induction 3%, followed by maintenance at 2% using a nose cone. The mouse was placed on a warm platform in the supine position to keep the body temperature around 37°C. The chest hair is removed using hair removal gel cream (Nair). The limbs are taped onto the metal EKG leads. Echo was performed using VisualSonic Vevo 2100 system with a 40 MHz transducer for cardiac imaging. In brief, by placing the transducer along the long-axis of LV, and directing to the right side of the neck of the mouse, two-dimensional LV long-axis is obtained. Then the transducer is rotated clockwise by 90°, and the LV short-axis view is visualized. 2D-guided LV M-mode at the papillary muscle level is recorded from either the short-axis view and/or the long-axis view. Trans-mitral inflow Doppler spectra are recorded in an apical 4-chamber view by placing the sample volume at the tip of the mitral valves. Echo images are downloaded and analyzed oﬄine using images analyzing software (Vevo 2100, 1.70, VisualSonic). At least three beats of imaging were measured and averaged for the interpretation of any given measurement. End-diastolic and end-systolic left ventricular internal diameters (LVIDd, LVIDs) were measured from the left ventricular short axis view with 2D orientated M-mode imaging. Left ventricular systolic function was estimated by fractional shortening (FS, %) according to the following formula: FS (%) = [(LVIDd – LVIDs)/LVIDd] × 100. Ejection fraction (EF) was calculated using the end-systolic and end-diastolic volumes as described (Stypmann et al., 2009).

### Cardiomyocyte Isolation From 3-Week Old Mice

Mice (3-week old) were sacrificed by Avertin overdose followed by cervical dislocation. Hearts were dissected. Ventricular myocytes were isolated using a modified method of a previously described protocol (Zhou et al., 2000). Briefly, excised hearts were mounted on a Langendorf apparatus and perfused with Ca2+-free Tyrode solution for 2 min at 37°C, followed by 6–10 min of perfusion with the same Tyrode solution with additional 20 μM CaCl_2_, 1 mg/ml collagenase (Worthington type II), and 0.13 mg/ml trypsin. The ventricles were placed in a plastic container containing 5 ml of same enzyme solution, and cut into 4 pieces. Ventricular tissues were gently triturated with a plastic transfer pipet to dissociate individual myocytes. Non-cardiomyocytes were depleted by centrifugation at 200 *g* for 30 s. Cardiomyocytes were re-suspended in the same Tyrode solution with addition of 125 μM CaCl_2_ and 5 mg/ml BSA. Cardiomyocytes were then pelleted at 200 g for 30 s and fixed with 4% PFA.

### RNA Purification and qRT-PCR Analysis

Quantitative real-time PCR (qRT-PCR) analysis was performed using Trizol isolated RNA, which was used to generate cDNA using random hexamer primers and SuperScript III RT (Invitrogen). qRT-PCR primer sequences are listed in Supplementary Table 1. SYBR green detection of amplification was performed using the StepOne Plus cycler (Applied Biosystems). Transcript expression values were generated with the comparative threshold cycle (Delta CT) method by normalizing to the expression of the 18S gene.

### Statistical Analysis

Data are presented as mean ± standard error of the mean (s.e.m.). Student’s *t*-test, one-way ANOVA and two-way ANOVA were used to calculate statistical significance. *P* values are depicted as follows: ^∗^*P* < 0.05; ^∗∗^*P* < 0.01; ^∗∗∗^*P* < 0.001; ^∗∗∗∗^*P* < 0.0001. Results with *P* > 0.05 were considered not significant (*n.s*.). All analyses were performed with GraphPad Prism 7.

## Results

### Cardiomyocytes Reactivate Cell Cycle and Become Binuclear During Early Postnatal Life

**Figure 1:**
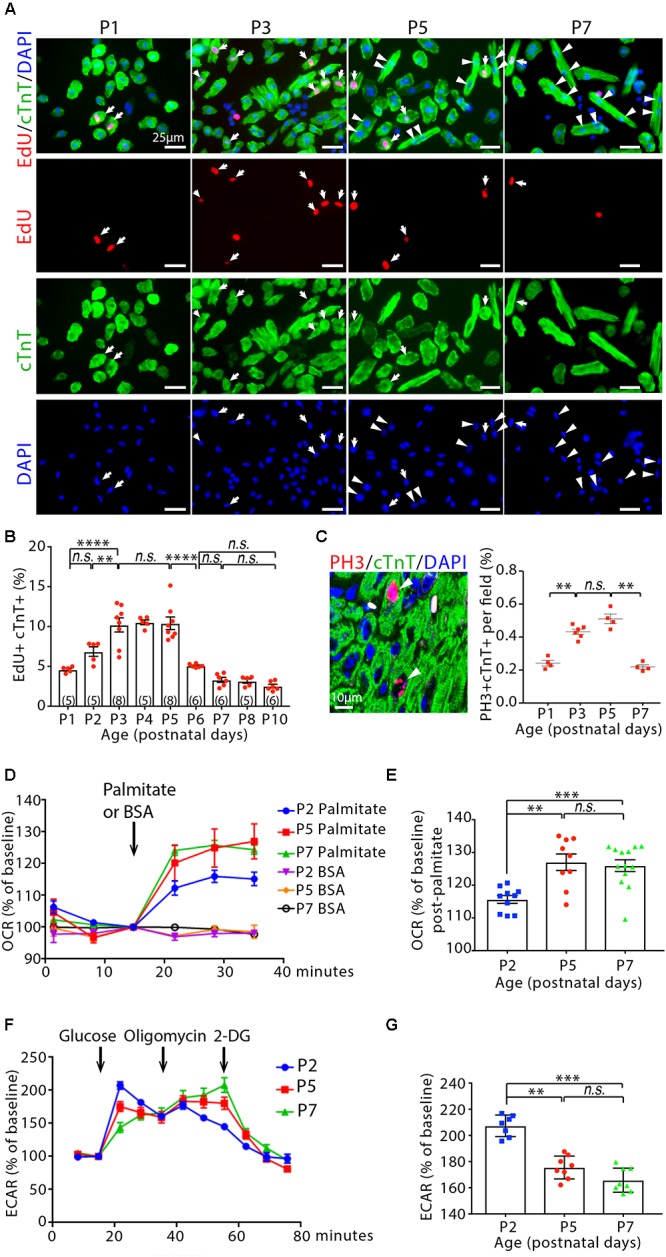
Cardiomyocyte cycling and metabolic profiling in infant mouse cardiomyocytes. **(A)** Isolated cardiomyocytes in DNA synthesis-phase were visualized by immunofluorescent microscopy using Click-iT EdU Alexa Fluor (red) and co-immunostaining with antibody against cardiac troponin T (cTnT, green). Arrows point to EdU+cTnT+ cells. Arrowheads point to binuclear cTnT+ cells. **(B)** Quantification of EdU+cTnT+ cells as percentage of total cTnT+ cells (∼1200 cTnT+ cells per sample). **(C)** Confocal images cardiomyocytes in mitotic phase as detected by co-immunostainings for phosphorylated histone H3 (PH3, red) and cTnT (green) on tissue sections, and quantification of PH3+cTnT+ cells as percentage of total cTnT+ cells analyzed per field. Arrows point to PH3+cTnT+ cells. **(D–G)** Isolated mouse cardiomyocytes from postnatal day 2 (P2), P5 and P7 heart ventricles were assessed with the Seahorse XF Analyzer. **(D)** Measurement and **(E)** quantification of mitochondrial oxygen consumption rate (OCR) with fatty acid stress test using palmitate versus BSA control. **(F)** Measurement and **(G)** quantification of extracellular acidification rate (ECAR) in the glycolysis stress assay. 2-DG (2-deoxyglucose) is a hexokinase inhibitor, which inhibits glycolytic pathway. *P* value was calculated using one-way ANOVA.

We measured total number of cardiomyocytes in the ventricular myocardium by enzymatic disaggregation and direct cell counting. The number of cardiomyocyte expanded continuously between 1-day-old (P1) and P10 mice (Supplementary Figure 1A). Notably, the rate of increase in cardiomyocytes number was highest within the first 3 days of age, with a 28.8 ± 2.0% increase from P1 to P3 (*P* < 0.05) (Supplementary Figure 1A). By contrast, the rate of increase was attenuated after P3 and the number of cardiomyocytes remained relatively constant between P3 and P5. This suggests a decline in cardiomyocyte hyperplasia growth. To determine DNA synthesis in cardiomyocytes, infant mice received a single intraperitoneal injection of 5-ethyl-2′-deoxyuridine (EdU) and were sacrificed after a 3-h labeling period. The frequency of EdU incorporation was determined on isolated ventricular cardiomyocyte by co-labeling with antibody against cardiac troponin T (cTnT) (Figure 1A). Consistent with our observations on cardiomyocytes number and previous reports (Soonpaa et al., 1996; Walsh et al., 2010; Ikenishi et al., 2012; Hirai et al., 2016), a transient increase of DNA synthesis was observed in infant cardiomyocytes, with a peak labeling index of ∼10% occurring between P3 and P5 (Figure 1B). The wave of increased cardiomyocyte cell cycle progression was also observed by immunostaining of sectioned hearts for EdU incorporation and the mitotic cell cycle marker phosphorylated histone H3 (PH3). The levels of EdU+Caveolin1+ and PH3+cTnT+ were highest in P3–P5 hearts, followed by a significant drop in P7 hearts (Supplementary Figures 1B,C). Accordingly, the DNA synthesis rate in cardiomyocytes increased from P1 to P5, reflecting the continuous progression of the cell cycle wave, whereas the total number of cardiomyocytes increased early but remained relatively constant from P3 to P5 (Supplementary Figure 1A). To determine whether cell division changes during postnatal life, we calculated the number of mono-, and bi-nuclear cardiomyocytes added to the ventricles by multiplying the average cardiomyocyte numbers by the percentages of cardiomyocyte that were mononucleate, or binucleate at these time points. The most striking change was the increase by (31.7 ± 1.2) × 10^4^ binucleated cardiomyocytes (4.5 ± 0.01-fold increase) that occurred from P3 to P5, followed by a further increase of (33.3 ± 1.5) × 10^4^ binucleated cardiomyocytes (1.8 ± 0.03-fold increase) from P5 to P7 (Supplementary Figure 1C). By contrast, (20.8 ± 0.9) × 10^4^ mononucleated cardiomyocyte (19.7 ± 0.1% decrease) were lost from P3 to P5. This is followed by a further loss of (24.2 ± 1.6) × 10^4^ mononucleated cardiomyocytes (28.6 ± 0.7% decrease) that was observed between P5 and P7 (Supplementary Figure 1D). Furthermore, we measured the expression of several mitosis-promoting genes in the cardiac ventricles from P1 to P10 and found significant increases in mRNA levels of these genes in both P3 and P5, with levels in P7 falling significantly compared to those in P1 (Supplementary Figure 1E). These data support the previous observations showing that the wave of postnatal cardiomyocyte cell cycle reactivation, marked by increased DNA synthesis, terminates before cell division and generates cardiomyocyte binucleation (Soonpaa et al., 1996; Ikenishi et al., 2012; Hirai et al., 2016).

### Cardiomyocyte Hypertrophic Growth and Maturation During Early Postnatal Life

We next assessed cardiomyocyte size as previously described (Chen et al., 2007). Freshly isolated cardiomyocytes were fixed prior to immunostaining for cTnT. Cells were then imaged and surface area was assessed using Image J. There was a ∼23.3 ± 4% increase in the size of cardiomyocyte from P1 to P3 (*P* < 0.01, Supplementary Figures 1F,G). Two dimensional surface area was further increased from 226.3 ± 29.2 μm^2^ in cardiomyocyte of P3 hearts to 347.12 ± 55.5 μm^2^ in the P5 hearts (53 ± 9% increase, *P* < 0.001). Cardiomyocyte size was further increased by 68.6 ± 8% in the P7 hearts (585.3 ± 41.9 μm^2^, *P* < 0.05, Supplementary Figures 1F,G). This result is consistent with the previous report that mammalian cardiomyocytes hypertrophic growth postnatally (Li et al., 1996).

Gene expression analysis on isolated heart ventricles showed that β-myosin heavy chain, *Myh7* (a marker of fetal cardiomyocytes) (Lyons et al., 1990; England and Loughna, 2013), mRNA decreased by ∼27% in P3 compared to P1 hearts (*P* < 0.05), ∼42% in P5 compared to P3 hearts (*P* < 0.05), and ∼60% in P7 compared to P5 hearts (*P* < 0.05). By contrast, *Myh6* (a marker of general cardiomyocytes) mRNA levels were not significantly changed (Supplementary Figure 1H). Moreover, the expression of *Mef2c* and *Nkx2.5*, the genes associated with fetal cardiomyocyte development (Olson, 2006; Li et al., 2016), followed the same pattern with *Myh7* gene expression and decreased significantly from P1 to P7 (Supplementary Figure 1H). Collectively, these results showed that the occurrences of cardiomyocyte hypertrophic growth and maturation were in parallel with cardiomyocyte cell cycle reactivation in infant mouse hearts.

### Cardiomyocyte Cell Cycle Reactivation and Hypertrophic Growth Are Accompanied by Metabolic Switch to Fatty Acid β-Oxidation

We used metabolic-flux analysis (with the Seahorse XF Analyzer) to assess mitochondrial respiration and anaerobic glycolysis by measuring the OCR and ECAR, respectively. Fatty acid stress test using palmitate revealed that P5 and P7 cardiomyocytes have greater OCR increase in response to palmitate than P2 cardiomyocytes (*P* < 0.01, Figure 1D,E). By contrast, a significant decrease was observed in the maximum ECAR in the glycolysis stress assay for cardiomyocytes at P5 and P7 compared to cardiomyocytes at P2 (Figure 1F,G). Together, these data suggest a shift away from glycolytic metabolism and toward fatty acid β-oxidation in infant cardiomyocytes between 2 and 5 days after birth, coinciding with cardiomyocyte cell cycle reactivation and hypertrophic growth.

### Inhibition of Cardiomyocyte Fatty Acid β-Oxidation Enhances Glycolysis and Maintains the Ability of Cardiomyocyte to Proliferate in Infant Mice

**Figure 2:**
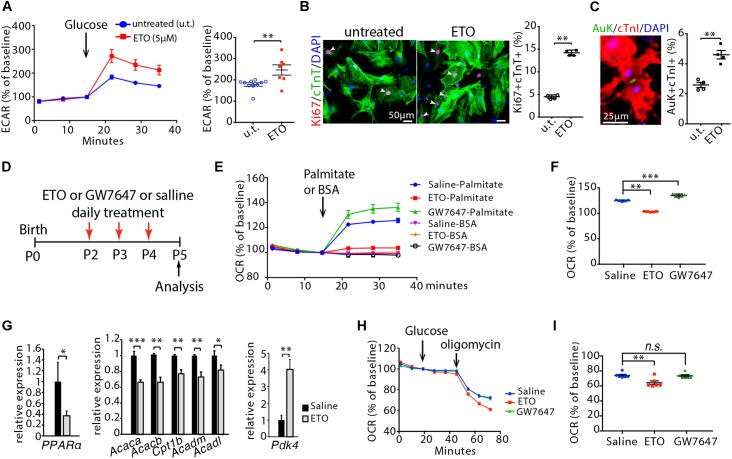
Effects of etomoxir (ETO) and GW7647 treatment on cardiomyocyte metabolism. **(A)** Isolated neonatal mouse cardiomyocytes (P1) were cultured with medium containing ETO (5 μM) for 48 h. ECAR was measured in cardiomyocytes using the Seahorse XF Analyzer. **(B)** Cardiomyocyte proliferation were visualized by co-immunostaining for Ki67 (red) and cTnT (green) on cultured cardiomyocytes, and quantification of Ki67+cTnT+ as percentage of total cTnT+ cells analyzed. Arrows point to Ki67+cTnT+ cells. **(C)** Cardiomyocytes in cytokinesis were visualized by co-immunostaining for Aurora B kinase (Auk, green) and cTnI (red) on cultured cardiomyocytes, and quantification of Auk+cTnI+ as percentage of total cTnI+ cells analyzed. **(D)** Schematic of experimental design for experiments performed in panels **(E–I)**. Infant mice were treated either with ETO or GW7647 or saline at P2, P3, and P4. Cardiomyocytes were isolated at P5 and processed for Seahorse analysis or gene expression analysis. **(E,F)** OCR was measured **(E)** and quantified in response to palmitate or BSA challenge **(F)**. **(G)** Expression of indicated genes by qRT-PCR analysis of the mRNA of isolated heart ventricles at P5 (*n* = 4–6 per group). **(H,I)** OCR was measured in isolated cardiomyocytes at P5 **(H)** and quantified in response to glucose challenge **(I)**. *P* value was calculated using Student’s *t*-test **(A–C, G)** and one-way ANOVA **(F, I)**.

We administered etomoxir (ETO), an inhibitor of carnitine palmitoyltransferase I (CPT1) (Lopaschuk et al., 1988), which is a key regulator of mitochondrial fatty acid uptake, in cultured neonatal mouse cardiomyocytes. A 48 h exposure of cardiomyocytes to ETO (5 μM) increased ECAR compared to untreated cardiomyocytes (Figure 2A), which indicates enhanced glycolytic metabolism in cardiomyocytes. ETO-treated cardiomyocytes exhibited higher proliferation index than untreated cardiomyocytes, as shown by the numbers of Ki67 (cell cycle marker) and cTnT (cardiomyocyte marker) double positive cells (Ki67+/cTnT+) as well as Aurora B kinase (Auk, cytokinesis marker) and cTnI (cardiomyocyte marker) double positive cells (Auk+/cTnI+) (*P* < 0.01, Figure 2B,C).

Next, ETO was administered daily via intraperitoneal injections into infant mice at P2, P3, and P4. Ventricular cardiomyocytes were isolated at P5 and processed for Seahorse analysis. ETO administration decreased OCR in response to palmitate compared to saline-treated mice (Figure 2D–F). Analyses of mouse heart ventricles by qRT-PCR showed that ETO-treated mice had a significant decrease of fatty acid metabolism-associated genes (*PPARα, Acaca, Acacb, Cpt1b, Acadm, Acadl*) at P5 compared to the saline-treated mice (Figure 2G). Furthermore, ETO-treated hearts had significantly increased expression of pyruvate dehydrogenase kinase 4 (*Pdk4*), which inhibits catabolism of the glucose-derived pyruvate (Sugden and Holness, 1994) (Figure 2G). Analyses of glucose oxidation showed that ETO administration decreased oxygen OCR in response to glucose compared to saline-treated mice (Figure 2H,I). These results indicate that *in vivo* treatment of ETO reduced fatty acid β-oxidation and glucose oxidation in infant mouse hearts.

**Figure 3:**
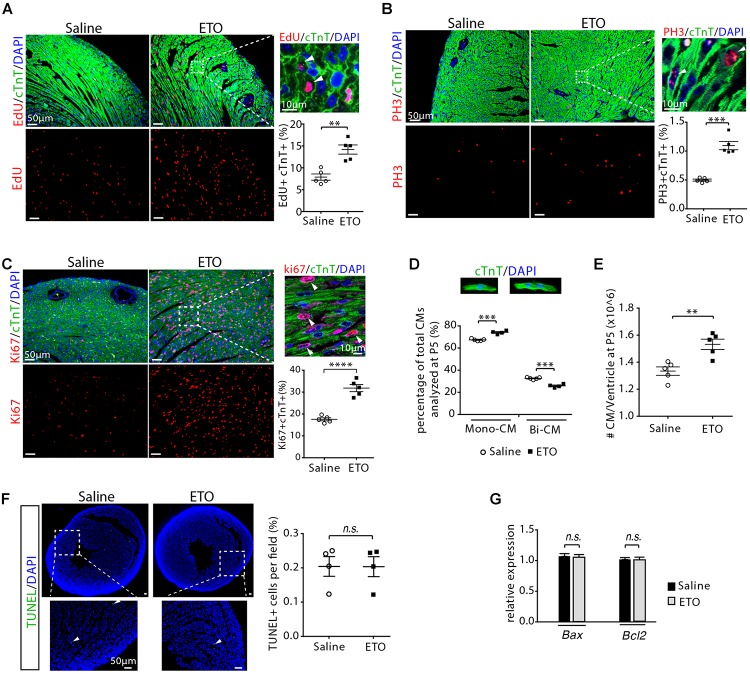
Cardiomyocyte proliferation with etomoxir (ETO) treatment. **(A)** Cardiomyocytes in DNA synthesis-phase were detected by using Click-iT EdU Alexa Fluor (red) and co-immunostaining with antibody against cTnT (green) on tissue cross sections. Quantification of EdU+cTnT+ cells as percentage of total cTnT+ cells analyzed per field. Arrows point to EdU+cTnT+ cells. **(B)** Cardiomyocytes in mitotic phase were detected and quantified by immunostaining for PH3 (red) and cTnT (green) on tissue longitudinal sections. Arrows point to PH3+cTnT+ cells. **(C)** Cardiomyocyte proliferation were visualized by co-immunostaining of heart sections (P5) for Ki67 (red) and cTnT (green). Graph on the right showing quantification of Ki67+cTnT+ as percentage of total cTnT+ cells analyzed per field. Arrows point to Ki67+cTnT+ cells. **(D)** Percentage of mononuclear (Mono-CM) and binuclear (Bi-CM) cardiomyocytes in the heart ventricles of infant mice at P5. **(E)** Total number of cardiomyocytes in heart ventricles in infant mice at P5. **(F)** Immunostaining and quantification of TUNEL (green) and DAPI (blue) in P5 heart sections. Arrows point to TUNEL+ nuclei. **(G)** Expression of *Bax* and *Bcl2* by qRT-PCR analysis of the mRNA of isolated heart ventricles at P5 (*n* = 4 per group). *P* value was calculated using Student’s *t*-test **(A–C, E–G)** and two-way ANOVA **(D)**.

To examine cell proliferation, infant mice, either treated with ETO or saline at P2, P3, and P4, were pulsed with EdU for 3 h at P5. Consistent with the *in vitro* observations, we found that treatment with the ETO *in vivo* increased cell cycle activity in cardiomyocytes (EdU+/cTnT+, PH3+/cTnT+, Ki67+/cTnT+) (Figure 3A–C). To determine whether cell division was altered in ETO-treated hearts, we isolated cardiomyocytes from P5 heart ventricles and counted the number of mononucleated and binucleated cardiomyocytes. Notably, ETO treatment inhibited cardiomyocyte binucleation prominence and resulted in a 31 ± 8.9% increase in total number of cardiomyocytes compared to saline-treated mice (Figure 3D,E). TUNEL staining of heart sections showed no significant difference in the number of apoptotic cells between ETO- and saline-treated heart at P5 (Figure 3F). Cardiac expression of apoptosis-associated genes (*Bax, Bcl2*) was not affected at P5 (Figure 3G).

### Inhibition of Fatty Acid β-Oxidation Delays Cell Cycle Exit, Hypertrophic Growth and Maturation in Infant Mouse Cardiomyocytes

**Figure 4:**
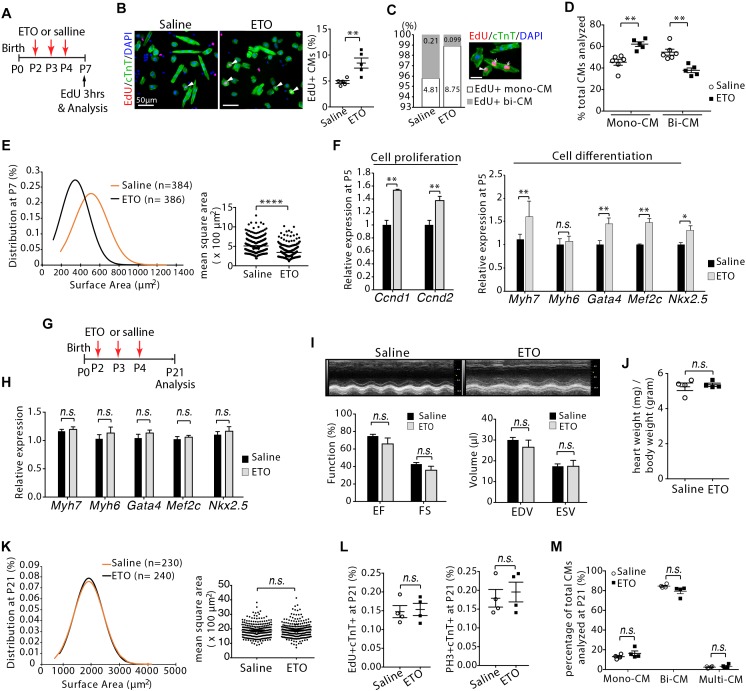
Cardiomyocyte size growth and maturation with etomoxir (ETO) treatment. **(A)** Schematic of experimental design for experiments performed in panels **(B–E)**. **(B)** Isolated cardiomyocytes in DNA synthesis-phase were visualized by immunofluorescent microscopy using Click-iT EdU Alexa Fluor (red) and co-immunostaining with antibody against cTnT (green). Arrowheads point to EdU+cTnT+ cells. Quantification of EdU+cTnT+ cells as percentage of total cTnT+ cells (∼940 cTnT+ cells per sample). Scale bars: 50 μm. **(C)** The percentage of mononucleated EdU+ cardiomyocytes (Mono-CM) and binucleated EdU+ cardiomyocytes (Bi-CM) at P7. Arrowheads point to mononuclear EdU+cTnT+ cells. Arrows point to binuclear EdU+cTnT+ cells. Scale bar: 25 μm. **(D)** Percentage of mononuclear (Mono-CM) and binuclear (Bi-CM) cardiomyocytes in the heart ventricles of infant mice at P7. **(E)** The frequency distribution and mean square areas of the surface area of cardiomyocytes isolated from P7 mouse heart ventricles. **(F)** Expression of indicated genes by qRT-PCR analysis of the mRNA of isolated heart ventricles at P5 (*n* = 5 per group). **(G)** Schematic of experimental design for experiments performed in panels **(H–M)**. **(H)** Quantification of gene expression by qRT-PCR analysis of the mRNA of isolated heart ventricles at P21 (*n* = 4 per group). **(I)** Cardiac function in mice evaluated by echocardiography at P21 (*n* = 4 per group). EF, ejection fraction; FS, fractional shortening; EDV, end-diastolic volume; ESV, end-systolic volume. **(J)** Heart weight-to-body weight ratios at P21. **(K)** The frequency distribution and mean square areas of isolated ventricular cardiomyocytes at P21. **(L)** Quantification of EdU+cTnT+ and PH3+cTnT+ as percentage of total cTnT+ cells analyzed on heart sections at P21. **(M)** Percentage of mononuclear (Mono-CM), binuclear (Bi-CM) and multinuclear (Multi-CM) cardiomyocytes in the heart ventricles of adult mice at P21. *P* value was calculated using Student’s *t*-test.

To determine whether cardiomyocyte proliferation was maintained at later stage, infant mice were treated with ETO at P2, P3, and P4 and pulsed with EdU for 3 h at P7 (Figure 4A). Increased DNA synthesis was continuously observed in cardiomyocytes of P7 mice as determined by quantification of the EdU+cTnT+ cells isolated from heart ventricles (1.76 ± 0.2-fold higher in ETO-treated mice compared to saline-treated group, *P* < 0.01, Figure 4B). There was a ∼53% reduction in the percentage of binucleated EdU+cTnT+ cells in ETO-treated hearts compared to saline-treated hearts (0.1 ± 0.01% vs. 0.21 ± 0.11%, *P* < 0.05, Figure 4C). Furthermore, ETO treatment led to a reduction in cardiomyocyte binucleation compared to saline treatment (31 ± 1.6%, *P* < 0.01, Figure 4D). These data indicated that ETO treatment maintained the ability of cardiomyocyte to proliferate in infant mice.

Since cardiomyocyte proliferation is associated with the onset of hypertrophic growth during early postnatal life (Li et al., 1996), we examined the effect of ETO treatment on the size of cardiomyocytes. Analysis of two dimensional surface area of isolated cardiomyocytes from heart ventricles revealed that cardiomyocytes from ETO-treated mice had decreased cell surface area compared to those from saline-treated mice (395.05 ± 55.5 μm^2^ vs. 585.28 ± 41.9 μm^2^, *P* < 0.0001, Figure 4E). qRT-PCR analysis using isolated heart ventricles at P5 from mice treated with ETO compared to saline-treated mice showed significantly increased expression of genes associated with positive regulation of cell proliferation (*Ccnd1, Ccnd2*), as well as fetal cardiomyocyte program-associated genes, including *Myh7, Gata4, Mef2c, and Nkx2.5* (Figure 4F). These results indicated that ETO-mediated inhibition of fatty acid β-oxidation reduced cardiomyocyte hypertrophic growth and maturation. Because it has been shown that prolonged less maturation in cardiomyocytes results in decreased cardiac function (Tian et al., 2015), we performed an additional and separate blinded study to assess whether the effects of ETO on the hearts could be resolved at later time point. Those observations were completely absent in P21 hearts, as the expression of those genes was equivalent between saline and ETO-treated mice (Figure 4G,H). Echocardiographic analysis showed normal cardiac function in ETO-treated mice compared to the saline-treated mice (Figure 4I). The ratio of heart weight to body weight was similar between saline and ETO-treated mice (Figure 4J). The size of ventricular cardiomyocytes isolated from ETO-treated mice was similar to that of saline-treated mice (Figure 4K). Moreover, cell cycle activity (EdU+/cTnT+, PH3+/cTnT+) and binucleation were similar between ETO- and saline-treated mice (Figure 4L,M).

Taken together, these results indicated that ETO-mediated inhibition of fatty acid β-oxidation in infant mouse hearts maintained the ability of cardiomyocyte to proliferate. However, cardiomyocyte hypertrophic growth and maturation were reduced upon ETO treatment. Those effects of ETO waned over time, as seen by the similar level of cardiomyocyte cell cycle activity, cell size and maturation in the hearts at weaning age. We thus conclude that ETO-mediated inhibition of fatty acid β-oxidation in infant mouse heart delayed cardiomyocyte cell cycle exit and hypertrophic growth.

### Activation of PPARα-Mediated Fatty Acid β-Oxidation Promotes Cell Proliferation Rate and Hypertrophic Growth, but Does Not Alter Total Cell Numbers in Infant Mouse Cardiomyocytes

**Figure 5:**
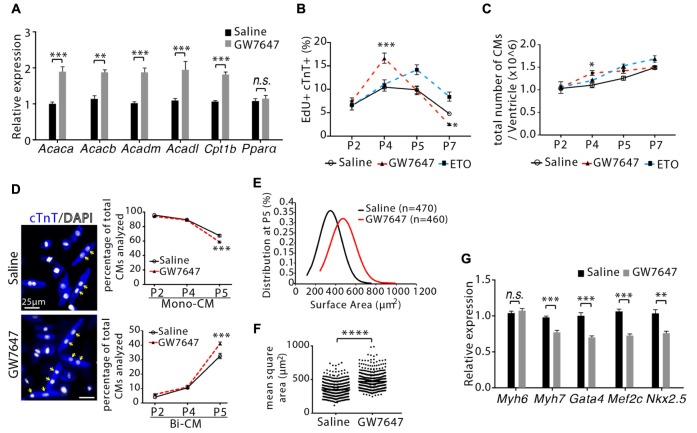
Cardiomyocyte proliferation and hypertrophic growth in GW7647-treated infant mouse hearts. **(A)** Infant mice were treated either with GW7647 or saline at P2, P3 and P4. Quantification of genes associated with fatty acid metabolism by qRT-PCR analysis of the mRNA of isolated heart ventricles at P5 (*n* = 4 per group). **(B)** Quantification of EdU+cTnT+ cells as percentage of total cTnT+ cells isolated from heart ventricles at indicated time points (*n* = 6 per group, ∼1000 cTnT+ cells per sample). **(C)** Quantification of total number of cardiomyocyte isolated from heart ventricles at indicated time points (*n* = 4–8 per group). **(D)** Representative images of isolated cardiomyocytes from P5 heart ventricles and quantification of the percentage of mononucleated (Mono-CM) and binucleated (Bi-CM) cardiomyocytes (*n* = 4–6 per group, ∼1000 cTnT+ cells per sample). Arrows point to Bi-CM. cTnT (blue). DAPI (gray). **(E,F)** Ventricular cardiomyocytes were isolated at P5 and measured for surface area. Quantitative analyses represent frequency distribution **(E)** and mean square areas **(F)** of the surface area of cardiomyocytes. **(G)** Quantification of genes associated with cardiomyocyte maturation by qRT-PCR analysis of the mRNA of isolated heart ventricles at P5 (*n* = 4–5 per group). *P* value was calculated using Student’s *t*-test **(A, F,G)**, one-way ANOVA **(D)** and two-way ANOVA **(B,C)**.

To promote fatty acid β-oxidation, we treated infant mice via intraperitoneal injections of GW7647, a highly specific PPARα agonist that increases fatty acid uptake and β-oxidation (Kersten et al., 1999). Using a similar approach as ETO treatment, infant mice were treated with GW7647 at P2, P3 and P4, and analyzed at P5 (Figure 2D). GW7647 administration significantly increased OCR in response to palmitate challenge in the isolated ventricular cardiomyocytes compared to saline-treated mice (Figure 2E). Analysis of the expression of PPARα target genes (*Acaca, Acacb, Acadm, Acadl, Cpt1b)*, which regulate fatty acid metabolism, by qRT-PCR showed a significant increase in the hearts of GW7647-treated mice compared to those of saline-treated mice (Figure 5A). Analyses of glucose oxidation showed that there was no significant difference in OCR in response to glucose between GW7647- and saline-treated cardiomyocytes (Figure 2H,I). These results indicated that *in vivo* treatment of GW7647 promoted fatty acid metabolism and β-oxidation in infant mouse heart.

We next evaluated the effect of GW7647-mediated PPARα activation on cardiomyocyte proliferation. Mice were pulsed with EdU for 3 h before harvesting tissues. DNA synthesis was quantified in isolated ventricular cardiomyocytes by visualizing EdU labeled cells that were co-immunostained with cardiomyocyte marker (cTnT). GW7647 treatment led to increased number of cardiomyocyte incorporating EdU (EdU+/cTnT+ cells) at P4 compared to saline or ETO treatment (16.6 ± 1.1% vs. 10.45 ± 0.8% for saline or 11.06 ± 0.6% for ETO, *P* < 0.001, Figure 5B). Quantification of the total number of ventricular cardiomyocytes showed that GW7647 treatment led to a 24 ± 5.9% increase in the total number of cardiomyocytes compared to saline or ETO treatment at P4 (*P* < 0.05, Figure 5C). However, the effect of GW7647 on cardiomyocyte proliferation became reversed over time, as seen by a quick fall in the number of EdU+/cTnT+ cells by P7 in mice with GW7647 treatment at P2, P3 and P4 (Figure 5B). Furthermore, the total number of cardiomyocytes was similar between GW7647-treated heart and saline-treated heart at P7 (Figure 5C). To determine whether GW7647-mediated PPARα activation affected cardiomyocyte hypertrophic growth and maturation, we examined cardiomyocytes binucleation, cell size and gene expression. Notably, the percentage of binucleated cardiomyocytes at P5 was ∼26% higher in GW7647-treated heart than saline-treated heart (41.2 ± 0.7% vs. 32.5 ± 1.1%, *P* < 0.001, Figure 5D). Assessment of cardiomyocyte size by quantifying two-dimensional surface area of isolated cardiomyocytes from P5 heart ventricles showed that GW7647 treatment led to increased cardiomyocyte size compared to saline treatment (472.6 ± 12.1 μm^2^ vs. 347.1 ± 16.9 μm^2^, *P* < 0.0001, Figure 5E,F). qRT-PCR analysis showed GW7647-treated hearts had a significant decrease in the expression of fetal cardiomyocyte program genes (*Myh7, Gata4, Mef2c, Nkx2.5*) compared with saline-treated hearts (Figure 5G), suggesting enhanced cardiomyocytes maturation in GW7647-treated hearts.

**Figure 6:**
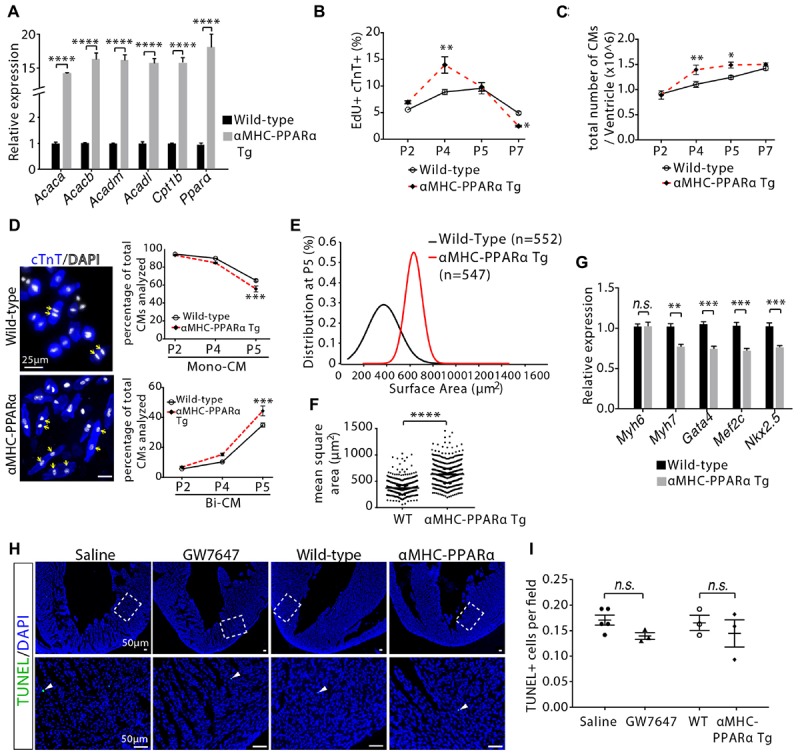
Cardiomyocyte proliferation and hypertrophic growth in αMHC-PPARα transgenic (Tg) hearts. **(A)** Quantification of genes associated with fatty acid metabolism by qRT-PCR analysis of the mRNA of isolated heart ventricles at P5 (*n* = 4 per group). **(B)** Quantification of EdU+cTnT+ cells as percentage of total cTnT+ cells isolated from heart ventricles at indicated time points (*n* = 6 per group, ∼1000 cTnT+ cells per sample). **(C)** Quantification of total number of cardiomyocyte isolated from heart ventricles at indicated time points (*n* = 4–9 per group). **(D)** Representative images of isolated cardiomyocytes from P5 heart ventricles and quantification of the percentage of mononucleated (Mono-CM) and binucleated (Bi-CM) cardiomyocytes (*n* = 4–6 per group, ∼1000 cTnT+ cells per sample). Arrows point to Bi-CM. cTnT (blue). DAPI (gray). **(E,F)** The frequency distribution **(E)** and mean square areas **(F)** of the surface area of cardiomyocytes isolated from αMHC-PPARα transgenic and wild-type (WT) heart ventricles at P5. **(G)** Quantification of genes associated with cardiomyocyte maturation by qRT-PCR analysis of the mRNA of isolated heart ventricles at P5 (*n* = 4–5 per group). **(H,I)** Immunostaining **(H)** and quantification **(I)** of TUNEL (green) and DAPI (blue) in P5 heart sections. The images in the second row are the enlargement of the bracketed regions shown in the top row. *P* value was calculated using Student’s *t*-test **(A,F,G,I)** and one-way ANOVA **(B–D)**.

In order to determine whether PPARα-mediated increase in fatty acid β-oxidation controlled cardiomyocyte proliferation and maturation in a cell-autonomous manner, we utilized a previously established transgenic mouse line, in which PPARα is overexpressed specifically in cardiomyocytes (αMHC-PPARα) (Finck et al., 2002). qRT-PCR analysis confirmed high level expression of PPARα and its target genes associated with fatty acid metabolism in P5 hearts (Figure 6A). Consistent with the findings observed with the GW7647 treatment, αMHC-PPARα mice showed increases in the number of EdU+/cTnT+ cells and the total number of cardiomyocytes in the P4 hearts (Figure 6B,C). By P7, the number of EdU+/cTnT+ cells in the αMHC-PPARα heart was significantly lower than that in the wild-type littermate (Figure 6B), and the total number of cardiomyocytes was similar between αMHC-PPARα heart and wild-type control (Figure 6C). Furthermore, αMHC-PPARα transgenic hearts at P5 exhibited enhanced cardiomyocyte hypertrophic growth and maturation, as evidenced by the significant increases in the percentage of binucleated cardiomyocytes and cell size (Figure 6D–F), as well as by the significant decreases in the expression of fetal cardiomyocyte program genes (*Myh7, Gata4, Mef2c, Nkx2.5*) compared with wild-type controls (Figure 6G). Cell apoptosis was not significantly changed in GW7647-treated and αMHC-PPARα animals compared to the control animals as determined by TUNEL staining on heart sections (Figure 6H,I).

Together, these findings indicated that activation of PPARα-mediated fatty acid β-oxidation in infant mouse heart initially increased cardiomyocyte proliferation rate by inducing more numbers of cardiomyocytes to enter the cell cycle and proliferate. As the cell cycle proceeded, PPARα-mediated fatty acid β-oxidation promoted cardiomyocyte enlargement (hypertrophic growth), maturation and, eventually, generated bi-nucleated cardiomyocytes. This accelerated cell hypertrophic growth and maturation induced by activation of fatty acid β-oxidation reduced cardiomyocyte proliferation, which ultimately resulted in no change in the total number of cardiomyocytes in P7 hearts.

### An Integrated Model of Fatty Acid β-Oxidation Effects on Cardiomyocyte Proliferation in Infant Mouse Heart

**Figure 7:**
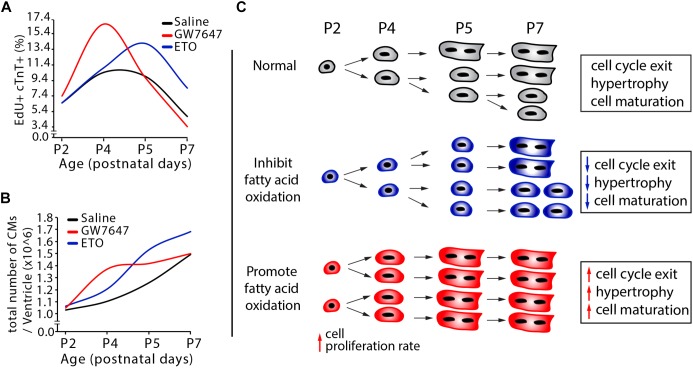
Effects of fatty acid oxidation on cardiomyocyte proliferation in infant mice. **(A,B)** Quantification of postnatal cardiomyocyte DNA synthesis and cell number expansion in mice with either GW7647, ETO or saline treatment. **(C)** The graphs depict the changes in cardiomyocyte proliferation, hypertrophic growth and maturation based on data obtained by DNA synthesis and cell counting analysis (Figure 3D–E, 5B–G).

Based on our data obtained by design-based stereology and by EdU labeling and cell counting analysis (Figure 5B,C), we established a quantitative model of DNA synthesis and total cell numbers in infant cardiomyocytes (Figure 7A,B). Inhibition of PPARα-mediated fatty acid β-oxidation delayed cardiomyocyte cell cycle exit. This was accompanied by decreased cardiomyocyte hypertrophic growth and maturation (Figure 7C). By contrast, in response to PPARα-mediated fatty acid β-oxidation, cardiomyocytes proliferation rate initially increased, reaching a maximum at P4 (Figure 7A,C). This was followed by accelerated cardiomyocyte hypertrophic growth and maturation induced by fatty acid β-oxidation, which led to cell cycle exit. As a consequence, activation of PPARα-mediated fatty acid β-oxidation does not alter the total number of cardiomyocytes in infant mice (Figure 7B).

## Discussion

Bioenergetic balance is a critical aspect for flawless operation of various biological systems and cellular processes. In this study, we have characterized an essential role for cardiac energy metabolism in regulating cardiomyocyte growth during early postnatal life. Cell proliferation, differentiation and metabolism maturation are three fundamental features of mammals. Interestingly, cell proliferation and metabolism have both been implicated in other biological processes such as regulation of pluripotent stem cell state and T cell activation in immune system (Cho et al., 2006; Chung et al., 2007, 2010; Gubser et al., 2013; Assmann and Finlay, 2016). Our findings go beyond association of proliferation with metabolic changes. We showed that the shift from glycolysis to fatty acid oxidation during postnatal life regulates cardiomyocytes proliferation and hypertrophic growth.

Opposite to what happened in embryonic hearts, which consume primarily glucose, fatty acids become the major fuel for ATP synthesis in cardiomyocytes of adult hearts (Lopaschuk and Jaswal, 2010). Cardiomyocytes proliferate rapidly during fetal life but lose their ability to proliferation soon after birth (Ahuja et al., 2007). However, before terminal withdrawal from the cell cycle, cardiomyocytes undergo another round of cell cycle during early postnatal life in mice, resulting in binucleated cardiomyocytes and increased cell size. Notably, those changes coincide with the metabolic shift from glycolysis to fatty acid β-oxidation. How and whether cardiac metabolic switch from glycolysis to fatty acid β-oxidation contributes to the cardiomyocyte proliferation and hypertrophic growth in normal development and physiology settings remained unanswered. Earlier studies demonstrated that in the early newborn period, the heart relies predominantly on glycolysis and lactate oxidation as sources of ATP (Lopaschuk and Spafford, 1990; Li et al., 1996 ; Makinde et al., 1998). Fatty acid β-oxidation rates remain low, providing less than 15% of the heart’s ATP requirements, due in part to an inhibition of mitochondrial fatty acid uptake (Lopaschuk et al., 1991; Li et al., 1997b). However, within days of neonatal life, a dramatic increase in fatty acid β-oxidation with a parallel decrease in glycolytic rates occur. By P7, glycolysis decreases further and provides less than 10% of total ATP production^5^. Thus, to meet the rigors of postnatal life, the heart undergoes a neonatal metabolic maturation that involves a switch concordant with a dramatic increase in mitochondrial functional capacity (Marin-Garcia et al., 2000; Lai et al., 2008). In this regard, we showed that, myocardial metabolic switch from glycolysis to fatty acid β-oxidation occurred within 5 days after birth and coincided with the burst of cardiomyocyte cell cycle reactivation and hypertrophic growth.

We found that ETO-mediated inhibition of fatty acid β-oxidation in infant mouse heart from postnatal days 2 to 4 maintained the capability of cardiomyocyte proliferation, prevented cardiomyocyte hypertrophic growth and maturation in the infant mouse hearts at P5 and P7. However, those effects of ETO waned over time, as seen by the similar level of cardiomyocyte cell cycle activity, cell size and maturation in the hearts at weaning age. These data suggest that ETO-mediated inhibition of fatty acid β-oxidation in infant mouse heart delayed cardiomyocyte cell cycle exit, hypertrophic growth and maturation. Previous studies have suggested the close association of cardiomyocyte cell cycle exit and cell maturation during postnatal life (Jonker et al., 2010; Paradis et al., 2014). Thus, the higher proliferating index in P5 and P7 hearts could be attributed to delayed cardiomyocyte maturation induced by ETO-mediated inhibition of fatty acid β-oxidation.

We found that activation of PPARα-mediated fatty acid β-oxidation initially enhanced cardiomyocyte proliferation in infant mice. Activation of PPARα-mediated fatty acid β-oxidation led to a higher rate on G0/G1 cell cycle entry and cardiomyocyte hyperplasia at P4. However, those observations were absent at P2 and P5. Previous reports from several research groups as well as our data showed that glycolysis is the major contributor to energy production in P1-P2 hearts (Lopaschuk and Spafford, 1990; Lopaschuk et al., 1991; Lopaschuk and Jaswal, 2010). Studies on isolated newborn hearts have shown that perfusion of neonatal heart under conditions optimal for fatty acid oxidation (palmitate) did not increase the contribution of fatty acid oxidation to overall energy production (Lopaschuk and Spafford, 1990; Lopaschuk et al., 1991), suggesting that metabolic machinery is not yet mature enough to use fatty acid as energy substrates for newborn heart. Thus, non-response to PPARα agonist in P2 heart may be due to lack of mature metabolic machinery. In addition, we found that activation of PPARα-mediated fatty acid β-oxidation in infant mouse heart promoted cardiomyocyte hypertrophic growth, maturation and generated binucleated cardiomyocytes at P5. Cardiomyocyte maturation has been shown to be closely associated with cell cycle exit, which is indicated by cytokinesis failure and cardiomyocyte binucleation during postnatal life (Jonker et al., 2010; Paradis et al., 2014). Thus, the quick drop in the proliferating index in P5 hearts may be caused by accelerated cardiomyocyte maturation induced by fatty acid β-oxidation.

Our results held one surprise that ran contrary to the conventional thinking on the fatty acid β-oxidation on cardiomyocyte proliferation. Studies of reactive oxygen species (ROS)-induced cardiomyocyte cell cycle arrest have suggested a reduction of mitochondrial-dependent oxidative stress as a strategy to promote cardiomyocyte proliferation (Puente et al., 2014). We found that activation of PPARα-mediated fatty acid β-oxidation initially promoted cardiomyocyte proliferation rate in infant mice. As the cell cycle proceeded, activation of PPARα-mediated fatty acid β-oxidation promoted cardiomyocytes hypertrophic growth, maturation and, eventually, generated binucleated cardiomyocytes. This accelerated cell hypertrophic growth and maturation induced by fatty acid β-oxidation reduced cardiomyocyte proliferation. As a consequence, activation of PPARα-mediated fatty acid β-oxidation did not alter the total number of cardiomyocytes in infant mice.

In summary, the current findings indicate that fatty acid β-oxidation plays an essential role in facilitating cardiomyocyte proliferation and hypertrophic growth in infant mouse heart. The infant mouse model represents a useful tool for modulation of metabolic pathways and for understanding the complex cellular and molecular mechanisms that dictate cardiomyocyte growth capacity.

## Data Availability

All datasets generated for this study are included in the manuscript and/or the Supplementary Files.

## Author Contributions

TC and DL performed majority of the experiments and participated in writing the manuscript. RLaCanna performed cell counting and quantification in infant mice. XZ performed P21 mouse cardiomyocytes isolation. BF advised on αMHC-PPARα animal model. TL performed cell counting and quantification. KD, XC, and RLu edited the manuscript. YT supervised all experiments and wrote the manuscript.

## Conflict of Interest Statement

The authors declare that the research was conducted in the absence of any commercial or financial relationships that could be construed as a potential conflict of interest.
