# Supplementary material for: Fatty Acid Oxidation Promotes Cardiomyocyte Proliferation Rate but Does Not Change Cardiomyocyte Number in Infant Mice
Source: Front Cell Dev Biol. 2019 Mar 22;7:42. doi: 10.3389/fcell.2019.00042 (PMC6440456; doi:10.3389/fcell.2019.00042)
Supplement: Supplementary file 1 [file Data_Sheet_2.PDF]

Supplemental Information:

Fatty acid oxidation promotes cardiomyocyte proliferation rate but does not change cardiomyocyte number in infant mice

Tongtong Cao[*], Daniela Liccardo[*], Ryan LaCanna, Xiaoying Zhang, Rong Lu, Brian N. Finck, Tani Leigh, Xiongwen Chen, Konstantinos Drosatos, Ying Tian

Correspondence should be addressed to: Ying Tian, email: ying.tian@temple.edu

Supplemental Figure 1

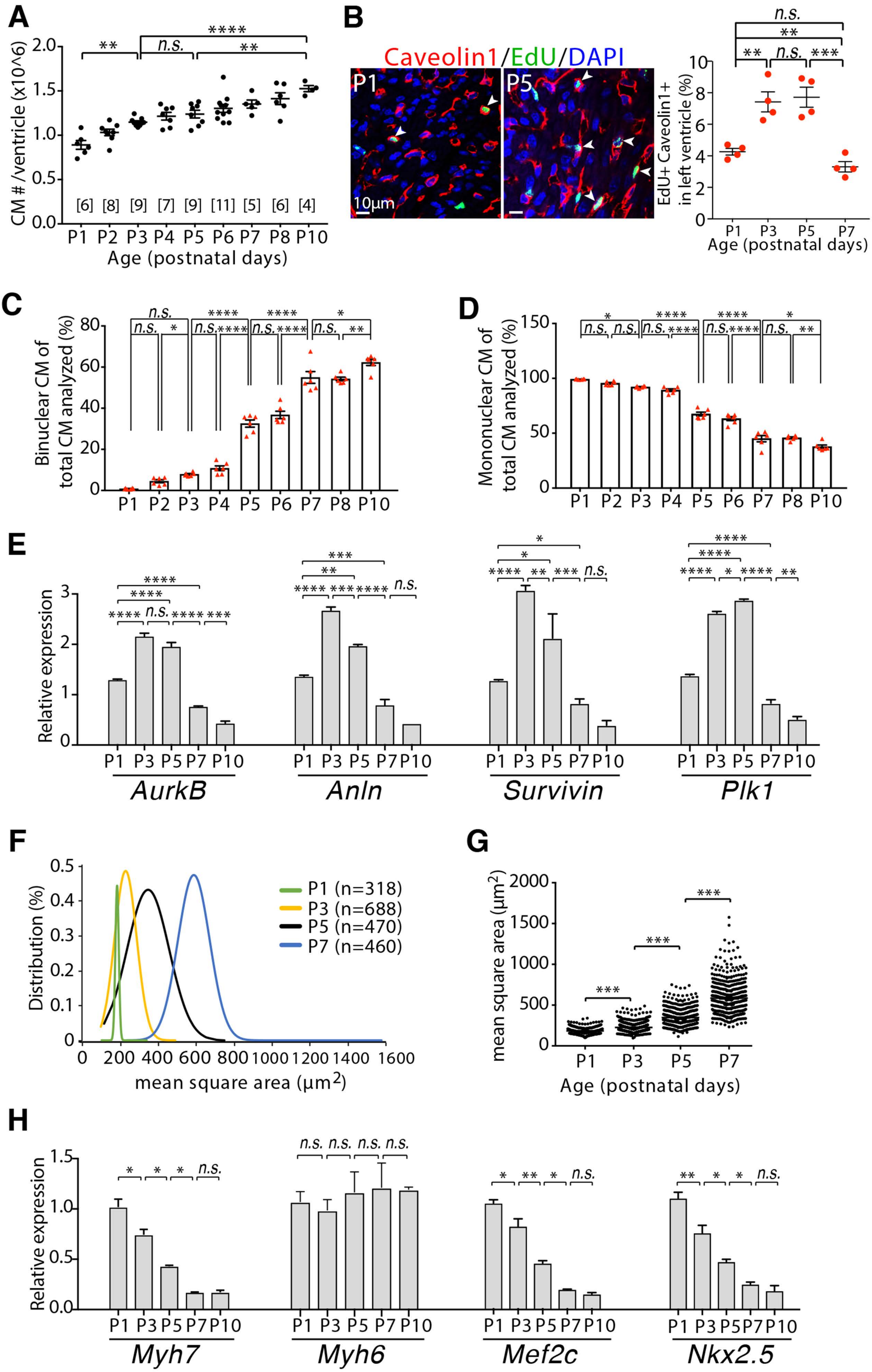

Supplemental Figure 1. Infant mouse hearts show evidence for cardiomyocyte cycling, hypertrophic growth and maturation. (**A**) Total number of cardiomyocytes (CM) in both cardiac ventricles of mice. (**B**) Confocal images of EdU+ cariomyocytes on tissue sections using Click-iT EdU Alexa Fluor (green) and co-immunostaining with antibody against caveolin1. Arrows point to EdU+Caveolin1+ cells. Graph on the right showing quantification of EdU+Caveolin1+ cells as percentage of total caveolin1+ cells analyzed per field. (**C** and **D**) Percentage of binuclear (**C**) and mononuclear (**D**) cardiomyocytes (CM) in the heart ventricles of infant mice. (**E** and **H**) Expression of indicated genes by qRT-PCR analysis of the mRNA of isolated heart ventricles at indicated time points (n= 5-6 per time point). (**F** and **G**) The frequency distribution (**F**) and mean square areas (**G**) of the surface area of cardiomyocytes isolated from infant mouse heart ventricles. *P* value was calculated using one-way ANOVA.

Supplemental Table 1. qRT-PCR primer sequences used in this study.

	Forward	Reverse
Myh6	CCACTTCTCCTTGGTCCACTATG	ACAAACCCACCACCGTCTCA
Myh7	AAGGGCCTGAATGAGGAGTAGCTC	GCAAAGGCTCCAGGTCTGA
Mef2c	GCCAGCACTG ACATGGATAAG	CCATTGAGGCCCTTCTTTCT
Gata4	CCGGGCTGTCATCTCACTATG	TTCAGAGCAGACAGCACTGGAT
Nkx2.5	TGACCCAGCCAAAGACCCT	CCATCCGTCTCGGCTTTGT
Acta1	TGAAGATGGGTTAAGCGGAG	TTCGTCGCACATGGTGTCTA
Nppa	GGGTAGGATTGACAGGATTGG	CTCCTTGGCTGTTATCTTCGG
Nppb	CTGAAGGTGCTGTCCCAGAT	CCTTGGTCCTTCAAGAGCTG
Acaca	CTGGGACAAAGAACCATCCA	ATAATCTGGATGCCCCCAAG
Acacb	CCGAGTTTGTCACTCGGTTT	GCATACACTTGACCGCAGC
Acadm	AGCTCTAGACGAAGCCACGA	TGAGCCTAGCGAGTTCAACC
Acadl	AACGTCTGGACTCCGGTTCT	CGGGTACTCCCACATGTACC
Cpt1b	TCTCCATGGGACTGGTCGAT	ACCATGCTGAGAAGTGCCTC
PParα	CAGTCCATCGGTGAGGAGAG	CTGGAAGCTGGAGAGAGGGT
Pdk4	CGTTCCTTCACACCTTCACC	GGTCAAGGAAGGACGGTTTT
Ccnd1	TTCCTCTCCAAAATGCCAGA	AGGGTGGGTTGGAAATGAAC
Ccnd2	GAACCTGGCCGCAGTCACCC	CGACGGCGGGTACATGGCAA
Bcl2	GTGGATGACTGAGTACCTGAAC	GAGACAGCCAGGAGAAATCAA
Bax	GTGGTTGCCCTCTTCTACTTT	CAGCCCATGATGGTTCTGAT
18S	TCAAGAACGAAAGTCGGAGG	GGACATCTAAGGGCATCAC
